# Genomics of Aminoglycoside Resistance in Pseudomonas aeruginosa Bloodstream Infections at a United States Academic Hospital

**DOI:** 10.1128/spectrum.05087-22

**Published:** 2023-05-16

**Authors:** Giancarlo Atassi, Rachel Medernach, Marc Scheetz, Sophia Nozick, Nathaniel J. Rhodes, Megan Murphy-Belcaster, Katherine R. Murphy, Arghavan Alisoltani, Egon A. Ozer, Alan R. Hauser

**Affiliations:** a Northwestern University Feinberg School of Medicine, Chicago, Illinois, USA; b Department of Pharmacy Practice, Pharmacometrics Center of Excellence, Chicago College of Pharmacy, Midwestern University, Downers Grove, Illinois, USA; c Department of Microbiology-Immunology, Northwestern University Feinberg School of Medicine, Chicago, Illinois, USA; d Pharmacometrics Center of Excellence, College of Graduate Studies, Department of Pharmacology, Midwestern University, Downers Grove, Illinois, USA; e Department of Medicine (Division of Infectious Diseases), Northwestern University Feinberg School of Medicine, Chicago, Illinois, USA; Emory University School of Medicine

**Keywords:** *Pseudomonas aeruginosa*, aminoglycosides, aminoglycoside modifying enzymes, resistance

## Abstract

Pseudomonas aeruginosa frequently becomes resistant to aminoglycosides by the acquisition of aminoglycoside modifying enzyme (AME) genes and the occurrence of mutations in the *mexZ, fusA1, parRS,* and *armZ* genes. We examined resistance to aminoglycosides in a collection of 227 P. aeruginosa bloodstream isolates collected over 2 decades from a single United States academic medical institution. Resistance rates of tobramycin and amikacin were relatively stable over this time, while the resistance rates of gentamicin were somewhat more variable. For comparison, we examined resistance rates to piperacillin-tazobactam, cefepime, meropenem, ciprofloxacin, and colistin. Resistance rates to the first four antibiotics were also stable, although uniformly higher for ciprofloxacin. Colistin resistance rates were initially quite low, rose substantially, and then began to decrease at the end of the study. Clinically relevant AME genes were identified in 14% of isolates, and mutations predicted to cause resistance were relatively common in the *mexZ* and *armZ* genes. In a regression analysis, resistance to gentamicin was associated with the presence of at least one gentamicin-active AME gene and significant mutations in *mexZ, parS,* and *fusA1*. Resistance to tobramycin was associated with the presence of at least one tobramycin-active AME gene. An extensively drug-resistant strain, PS1871, was examined further and found to contain five AME genes, most of which were within clusters of antibiotic resistance genes embedded in transposable elements. These findings demonstrate the relative contributions of aminoglycoside resistance determinants to P. aeruginosa susceptibilities at a United States medical center.

**IMPORTANCE** Pseudomonas aeruginosa is frequently resistant to multiple antibiotics, including aminoglycosides. The rates of resistance to aminoglycosides in bloodstream isolates collected over 2 decades at a United States hospital remained constant, suggesting that antibiotic stewardship programs may be effective in countering an increase in resistance. Mutations in the *mexZ, fusA1, parR, pasS,* and *armZ* genes were more common than acquisition of genes encoding aminoglycoside modifying enzymes. The whole-genome sequence of an extensively drug resistant isolate indicates that resistance mechanisms can accumulate in a single strain. Together, these results suggest that aminoglycoside resistance in P. aeruginosa remains problematic and confirm known resistance mechanisms that can be targeted for the development of novel therapeutics.

## INTRODUCTION

Pseudomonas aeruginosa is a high-priority pathogen with a propensity for causing severe opportunistic and nosocomial infections. Complicating the treatment of infections is significant intrinsic antimicrobial resistance (AMR) and a remarkable ability to mutate and horizontally acquire resistance determinants. As a result, many P. aeruginosa isolates are highly resistant to antibiotics, and one strain may carry numerous antibiotic resistance genes or alleles (1–3). Several of these multi-drug-resistant (MDR) and extensively drug-resistant (XDR) strains have disseminated globally and are referred to as “high-risk clones.” The 10 most common high-risk clones are sequence types (STs) 235, 111, 233, 244, 357, 308, 175, 277, 654, and 298 (4). High-risk clones account for substantial proportions of MDR P. aeruginosa isolates in some parts of the world and are a major health care threat.

Two approaches have been taken to address the challenges of antibiotic-resistant organisms. At the clinical level, many medical institutions have instituted antimicrobial stewardship programs. These programs have decreased inappropriate antibiotic use and nosocomial infections by resistant pathogens with good outcomes (5, 6). At the diagnostic level, molecular identification of resistance genes and alleles via nucleic acid amplification or whole genome sequencing has the potential to rapidly identify resistant and susceptible organisms, allowing for focused antimicrobial therapy (7). However, in many cases genomic resistance genes and alleles can only serve as surrogate markers of AMR and must be correlated to clinical phenotypes to validate their utility.

Aminoglycosides are commonly used to treat P. aeruginosa infections in individuals with cystic fibrosis and those with acute infections, and this bacterium deploys an array of resistance mechanisms against this class of antibiotics. Isolates can inactivate aminoglycosides via aminoglycoside modifying enzymes (AMEs), of which there are three primary families: phosphorylators (APH), acetylators (AAC), and adenylators (ANT) (8–10). Adding to this complexity, individual AMEs have variable activities against the different aminoglycosides. For example, AAC(6’)-Ia inactivates amikacin, whereas AAC(6’)-Ib’ inactivates gentamicin and tobramycin (8). Another mechanism of resistance is the overexpression of the MexXY-OprM efflux pump following mutational inactivation of in its repressor, *mexZ* (11). Other causes of aminoglycoside resistance include mutations in *fusA1*, which encodes the elongation factor EF-G1A; mutations in *armZ* (also called PA5471), which encodes an anti-repressor of *mexZ*, mutations in the two-component system ParRS, and modification of 16S rRNA molecules by methylases such as RmtA and RmtB (8, 12–15). As a result, aminoglycoside genotype/phenotype correlation studies with P. aeruginosa have been challenging (16–18).

We examined aminoglycoside resistance in P. aeruginosa bloodstream isolates at Northwestern Memorial Hospital (NMH) in Chicago, U.S. We examined changes in the prevalence of aminoglycoside resistance and AME genes over 2 decades and assessed the impact of AME genes on aminoglycoside resistance in these isolates. We also conducted an analysis of whether mutations in the genes *mexZ*, *fusA1*, *parRS*, and *armZ* predicted aminoglycoside resistance. Finally, we performed genomic characterization of an MDR isolate carrying five AME genes to examine the context of these resistance genes.

## RESULTS

### Aminoglycoside resistance in P. aeruginosa over time.

**FIG 1:**
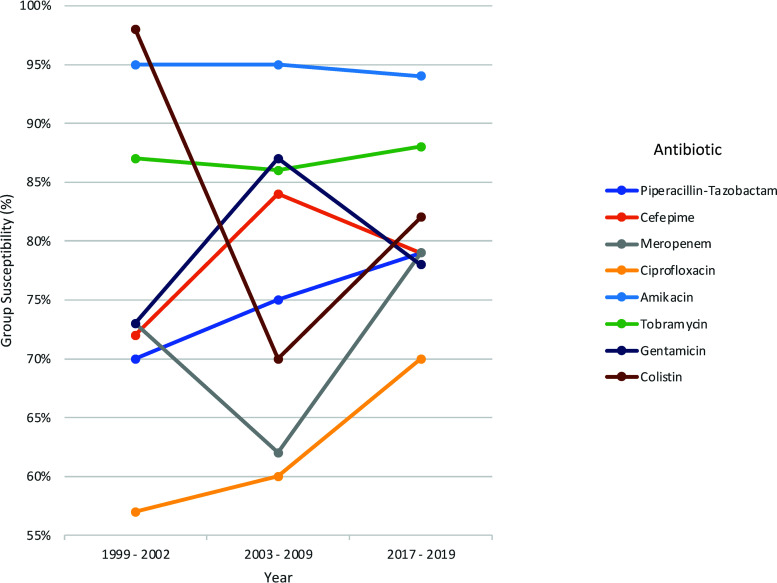
Susceptibility rates of P. aeruginosa isolates by antibiotic and time period.

We examined the aminoglycoside resistance of 227 P. aeruginosa bloodstream isolates obtained from three separate archived strain collections at NMH. The first archived collection contained 93 isolates from 1999–2002, the second contained 101 isolates from 2003–2009, and the third contained 33 isolates from 2017–2019 (Table S1). Susceptibility rates to the following commonly used aminoglycosides were measured: gentamicin, tobramycin, and amikacin. Susceptibility rates to amikacin were highest and remained stable across time, ranging from 94–95% (Table 1, Fig. 1). Susceptibility rates to tobramycin were somewhat lower (86-88%) but also remained stable. In contrast, susceptibility rates to gentamicin were lower still and more variable: 73% in 1999–2002, 87% in 2003–2009, and 78% in 2017–2019. Using the 2003–2009 collection as the referent range, a difference in gentamicin susceptibility existed between the 2003–2009 and the 2017–2019 periods (*P* = 0.02). Together, these results suggest P. aeruginosa isolates at NMH have remained stably susceptible to amikacin and tobramycin, whereas susceptibility rates to gentamicin have been variable. We compared these susceptibility rates to those of five other commonly used anti-pseudomonal antibiotics: piperacillin-tazobactam, cefepime, meropenem, ciprofloxacin, and colistin. For each of these except colistin, susceptibility rates did not significantly change across the three time periods (Table 1, Fig. 1). Susceptibility rates were lowest for ciprofloxacin, with only 57–70% of isolates being susceptible. For colistin, susceptibility rates were 98% in the first cohort, 70% in the second cohort, and 82% in the third cohort, with a statistically significant difference existing between the first and second cohorts (*P* < 0.001). These changes may reflect differences in the frequency of colistin use at NMH over time.

**TABLE 1:** Susceptibility of P. aeruginosa isolates to antibiotics by time period

	1999–2002	OR and *P*-Value	2003–2009	2017–2019	OR and *P*-value
	*n* = 93		*n* = 101	*n* = 33	
Piperacillin-Tazobactam					
Susc^*b*^	65 (70%)	OR = 0.80	75 (74%)	22 (79%)	OR = 1.27
NS	28 (30%)	*p* = 0.498	26 (26%)	6 (21%)	*p* = 0.641
Cefepime					
Susc	63 (72%)	OR = 0.47	85 (84%)	26 (79%)	OR = 0.70
NS	24 (28%)	*p* = 0.039	16 (16%)	7 (21%)	*p* = 0.479
Meropenem					
Susc	67 (73%)	OR = 1.62	63 (62%)	26 (79%)	OR = 1.71
NS	25 (27%)	*p* = 1.54	38 (38%)	7 (21%)	*p* = 0.08
Ciprofloxacin					
Susc	53 (57%)	OR = 0.87	61 (60%)	23 (70%)	OR = 1.51
NS	40 (43%)	*p* = 0.63	40 (40%)	10 (30%)	*p* = 0.34
Amikacin					
Susc	88 (95%)	OR = 0.95	93 (95%)	31 (94%)	OR = 0.83
NS	5 (5.4%)	*p* = 0.932	5 (5%)	2 (6.1%)	*p* = 0.83
Tobramycin					
Susc	81 (87%)	OR = 1.08	87 (86%)	28 (88%)	OR = 1.13
NS	12 (13%)	*p* = 0.85	14 (14%)	4 (12%)	*p* = 0.85
Gentamicin					
Susc	68 (73%)	OR = 0.40	88 (87%)	25 (78%)	OR = 0.53
NS	25 (27%)	*p* = 0.016	13 (13%)	7 (22%)	*p* = 0.22
Colistin					
Susc	86 (98%)	**OR = 18.4**	70 (70%)	27 (82%)	OR = 1.92
NS	2 (2%)	***p* < 0.001** ^*a*^	30 (30%)	6 (18%)	*p* = 0.19

a *P*-values bolded indicate significance as calculated by logistic regression. The referent range is 2003–2009.

b Not all numbers add up to the total number of isolates in each collection, as susceptibility data were unavailable for some isolates. Susc = Susceptible; NS = Nonsusceptible.

### MLSTs of P. aeruginosa isolates.

High-risk clones of P. aeruginosa are frequently resistant to antibiotics, including aminoglycosides, but little is known about whether these clones are increasing or decreasing in prevalence. We therefore analyzed the MLSTs of each isolate for the presence of high-risk clone STs. We were able to assign 209 of the 227 isolates to existing MLSTs. The remaining 18 isolates had exact identity matches for all seven housekeeping genes but did not correspond to known MLSTs, indicating that they are novel STs (Table S1). Seven of the identified STs (ST111, ST233, ST235, ST244, ST277, ST308, and ST298) were high-risk clones (4). Although not statistically significant (*P* = 0.053), the numerical prevalence of these high-risk clones decreased from 22 of 93 isolates (24%) in 1999–2002 to 21 of 101 (21%) in 2003–2009 to 2 of 33 isolates (6%) in 2017–2019.

### Prevalence of AME genes in P. aeruginosa isolates.

We next examined the whole genome sequences of each isolate for AME and methylase genes (Table 2). *aph(3′)-IIb*, which confers resistance to kanamycin, neomycin, butirocin, and seldomycin, was found in all isolates. Other AME genes were relatively rare. *ant(2″)-Ia* was the most common AME gene in the 1999–2002 cohort (8 isolates), followed by several *aadA* genes (members of the ANT(3″)-Ia family of adenylating AMEs). In the 2003–2009 cohort, the most common AME gene was *aadA6* (5 isolates); *ant(2″)-Ia* was again fairly prevalent (4 isolates). In the 2017–2019 cohort, only *aadA6*, *aadA10*, and *ant(2″)-Ia* genes were present (1 isolate each), perhaps due to the smaller size of this collection. Overall, *ant(2″)-Ia* was the most common of the AME genes conferring resistance to clinically used aminoglycosides (13 [6%] of isolates), as has been observed in other studies (8). None of the cohorts contained isolates with the *rmtA* or *rmtB* methylase genes. Excluding *aph(3′)-IIb*, the prevalence of isolates with at least one AME gene decreased from 16 of 93 isolates (17%) in the 1999–2002 cohort to 14 of 101 isolates (14%) in the 2003–2009 cohort, to 2 of 33 isolates (6%) in the 2017–2019 cohort. These differences were not statistically significant.

**TABLE 2:** AME genes present in P. aeruginosa isolates

No. of Isolates with AME Gene (% of Isolates)						
Gene	Protein	Activity^*a*^	1999–2002	2003–2009	2017–2019	Total
			(*n* = 93)	(*n* = 101)	(*n* = 33)	(*n* = 227)
*aac*(3)-*Id*	AAC(3)-Id	gent	1 (1%)	0 (0%)	0 (0%)	1 (0.4%)
*aac*(3)-*IIIb*	AAC(3)-IIIb	gent, tob, kan	0 (0%)	2 (2%)	0 (0%)	2 (0.9%)
*aac(6′)-Ib*	AAC(6’)-Ib	tob, kan	1 (1%)	3 (3%)	0 (0%)	4 (2%)
*aac(6′)-Ib'*	AAC(6’)-Ib’	gent, tob	3 (3%)	0 (0%)	0 (0%)	3 (1%)
*aac(6′)-II*	AAC(6)-II	gent, tob	1 (1%)	0 (0%)	0 (0%)	1 (0.4%)
*aac(6′)-IIc*	AAC(6’)-IIc	gent, tob, kan	0 (0%)	1 (1%)	0 (0%)	1 (0.4%)
*aadA1*	ANT(3″)-Ia	strep	0 (0%)	2 (2%)	0 (0%)	2 (0.9%)
*aadA2*	ANT(3″)-Ia	strep	4 (4%)	0 (0%)	0 (0%)	4 (2%)
*aadA6*	ANT(3″)-Ia	strep	4 (4%)	5 (5%)	1 (3%)	10 (4%)
*aadA7*	ANT(3″)-Ia	strep	0 (0%)	3 (3%)	0 (0%)	3 (1%)
*aadA10*	ANT(3″)-Ia	strep	4 (4%)	3 (3%)	1 (3%)	8 (4%)
*ant(2″)-Ia*	ANT(2″)-Ia	gent, tob	8 (9%)	4 (4%)	1 (3%)	13 (6%)
*aph(3′)-Ib*	APH(3′)-Ib	kan, neo	0 (0%)	1 (1%)	0 (0%)	1 (0.4%)
*aph(3′)-IIb*	APH(3′)-IIb	kan, neo	93 (100%)	101 (100%)	33 (100%)	227 (100%)
*aph(3″)-Ib*	APH(3″)-Ib	strep	1 (1%)	0 (0%)	0 (0%)	1 (0.4%)
*aph*(6)*-Id*	APH(6)-Id	strep	1 (1%)	0 (0%)	0 (0%)	1 (0.4%)
Isolates with at least 1 AME gene^*b*^			16 (17%)	14 (14%)	2 (6%)	32 (14%)

a Published activities of these AME genes against gentamicin (gent), tobramycin (tob), kanamycin (kan), neomycin (neo), and streptomycin (strep) are indicated (8, 9).

b Excluding aph(3’)-IIb.

### Associations between AME genes and aminoglycoside resistance.

We next examined whether individual or combinations of AME genes predicted phenotypic resistance to aminoglycosides. We focused on gentamicin and tobramycin because we did not identify genes encoding AMEs with activity against amikacin in our collection. For gentamicin and tobramycin, we attempted to perform logistic regressions to individually examine each of the AME genes predicted to have activity against these agents. However, due to the few isolates that contained each of the individual AME genes, estimating odds ratios by logistic regression was not possible. We instead collapsed all the AME genes with predicted activity against gentamicin or predicted activity against tobramycin into composite variables and performed regression analyses with gentamicin resistance or tobramycin resistance as the dependent variables. Statistically significant associations were found between the presence of at least one gentamicin-active AME gene and gentamicin resistance (OR 44.8, *P* < 0.001) as well as the presence of at least one tobramycin-active AME gene and tobramycin resistance (OR 36.2, *P* < 0.001).

### Associations between *mexZ* mutations and aminoglycoside resistance.

To further characterize possible mechanisms of resistance, we examined the *mexZ* gene for the presence of nonsynonymous mutations, frameshift mutations, or premature stop codons. We reasoned that these mutations had the potential to disrupt the function or expression of the MexZ repressor, which would lead to overexpression of the MexXY efflux pump. Since nonsynonymous mutations can have no effect, a small effect, or a large effect on the encoded protein, we used the program PROVEAN (32, 33) to identify those nonsynonymous mutations most likely to disrupt protein function. A total of 32 isolates had nonsynonymous mutations in *mexZ* that met this criterion (Table S1). An additional 15 isolates had frameshift mutations; none had premature stop codon mutations (Table S1). We combined the 32 nonsynonymous mutations, the frameshift mutations, and the premature stop codon mutations into a category we will hereafter refer to as “significant mutations.” We next performed logistic regression analysis to determine whether significant mutations were associated with nonsusceptibility to gentamicin or tobramycin. There was a statistical difference when comparing significant mutations in *mexZ* with gentamicin resistance (OR 3.5, *P* = 0.002) but not tobramycin resistance (OR 1.6, *P* = 0.33).

We also examined the intergenic region between *mexZ* and *mexX*, where the MexZ repressor binds (38), because polymorphisms in this region have been identified in aminoglycoside-resistant isolates (39). Twenty-two isolates contained at least one single nucleotide variant (SNV) in this region (Table S1), but due to the variability in the type and location of these SNVs we were unable to determine associations with aminoglycoside resistance.

### Associations between *fusA1* mutations and aminoglycoside resistance.

Naturally occurring mutations in the *fusA1* gene have been linked to enhanced resistance to aminoglycosides (40, 41). We therefore applied the same approach described for *mexZ* to *fusA1.* Three isolates had significant nonsynonymous mutations in *fusA1*, but no frameshift or premature stop codon mutations were observed (Table S1). There was a trend toward a statistical difference when comparing significant mutations in *fusA1* to gentamicin resistance (OR 8.4, *P* = 0.086) but not to tobramycin resistance (OR 3.3, *P* = 0.33).

### Associations between *parRS* mutations and aminoglycoside resistance.

Mutations in the two-component system *parRS* have also been associated with aminoglycoside resistance (8, 12–15). We examined these genes and found no frameshift mutations or premature stop codon mutations in *parR* but four nonsynonymous mutations that passed the PROVEAN criterion (Table S1). The presence of a significant mutation in *parR* was statistically associated with gentamicin resistance (OR 6.4, *P* = 0.046) but not tobramycin resistance (OR 1.7, *P* = 0.66). Findings were similar in *parS* except that there were 13 nonsynonymous mutations that passed the PROVEAN criterion (Table S1). The presence of a significant mutation in *parS* was statistically associated with gentamicin resistance (OR 4.5, *P* = 0.013) but not tobramycin resistance (OR 1.3, *P* = 0.72).

### Associations between *armZ* mutations and aminoglycoside resistance.

Mutations in *armZ* as well as its upstream region, involved in ribosome-dependent translational attenuation of *armZ*, have been associated with over-expression of *mexXY* and aminoglycoside resistance (42, 43). We therefore examined the *armZ* sequence and its 367 bp intergenic upstream region. The *armZ* gene did not contain frameshift or premature stop codon mutations but did contain many nonsynonymous mutations (Table S1). One of these, causing a H182Q substitution, had a significant PROVEAN score but was found in 77% of the isolates. This mutation was deemed a polymorphism between the reference strain, PAO1, and many of the clinical isolates and was excluded from further analyses. Twenty isolates contained other nonsynonymous mutations that were significant by the PROVEAN criterion. The presence of at least one of these mutations was statistically associated with gentamicin resistance (OR 3.1, *P* = 0.004) and tobramycin resistance (OR 2.6, *P* = 0.035).

We also examined the intergenic region upstream of *armZ*. Seventy-nine isolates contained at least one single nucleotide variant (SNV) in this region (Table S1), but due to the variability in the type and location of these SNVs we were unable to determine associations with aminoglycoside resistance.

### Comprehensive regression analysis.

We next performed a regression analysis taking into account all of the genes in the preceding analyses. In this model, the following genes (with odds ratios, *P*-values) were statistically associated with gentamicin resistance: *parS* (10.7, <0.001), *fusA1* (17.2, 0.027), *mexZ* (4.5, 0.003), and the presence of at least one AME gene predicted to be active against gentamicin (54.7, <0.001). Only the presence of at least one AME gene predicted to be active against tobramycin (45.8, <0.001) was statistically associated with tobramycin resistance.

### Association of aminoglycoside resistance determinants with high-risk clones.

**FIG 2:**
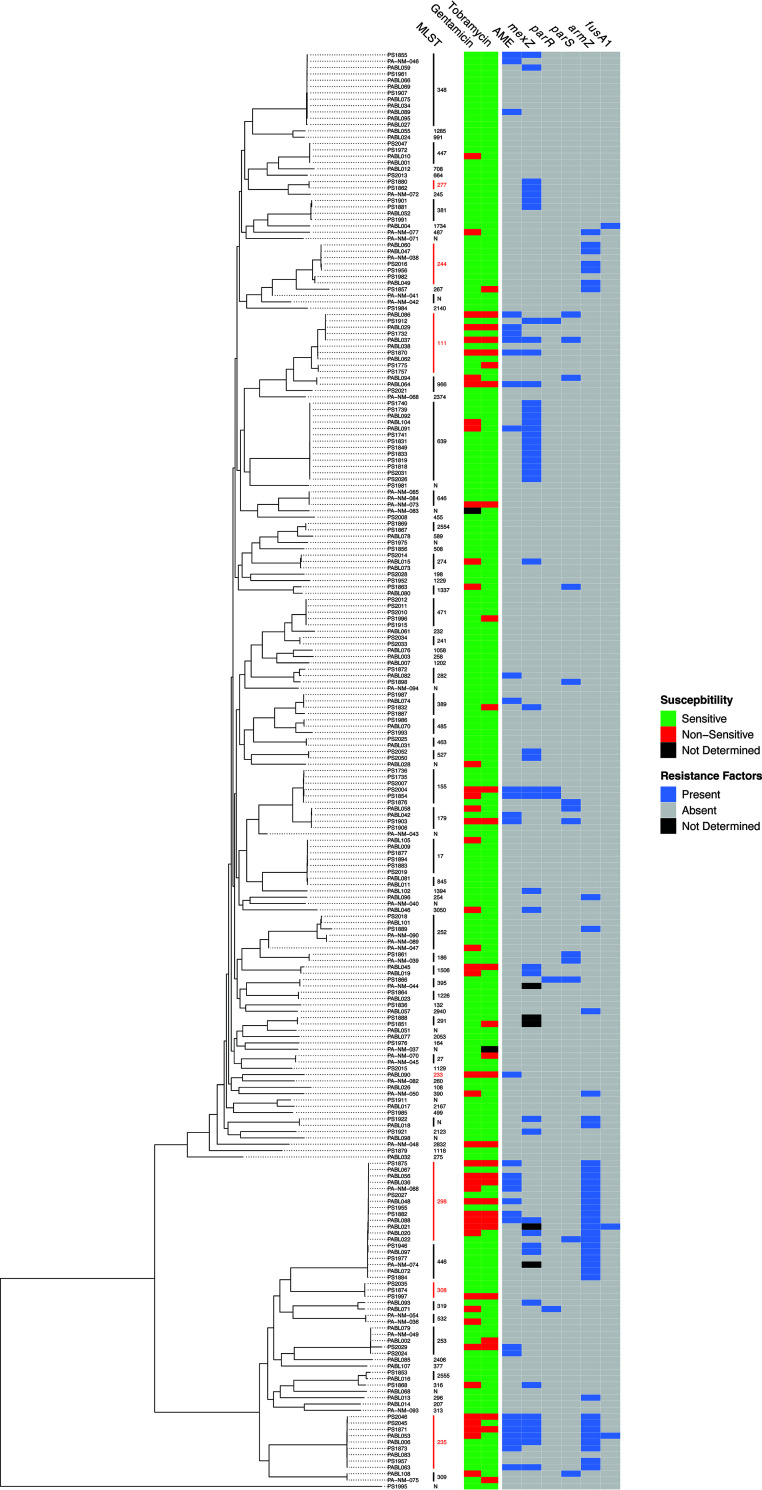
Phylogenetic distribution of aminoglycoside resistance determinants. A core genome phylogenetic tree was generated from the genomes of the 227 isolates and compared to the presence or absence of AME genes and significant mutations in *mexZ*, *parR*, *parS*, *armZ*, and *fusA1.* MLSTs and susceptibilities to gentamicin and tobramycin are also indicated. STs associated with high-risk clones are shown in red.

We next examined the phylogenetic distribution of AME genes and aminoglycoside resistance mutations among the isolates used in this study. A core genome phylogenetic tree was generated, and the presence of resistance determinants across the tree was plotted (Fig. 2). Resistance determinants were found throughout the tree but were nearly uniformly present in some STs, suggesting vertical transmission within that ST (e.g., *mexZ* mutations in ST639, *armZ* mutations in ST446). AME genes were frequently found in some isolates from an ST but not others, suggesting ongoing horizontal transmission (e.g., ST348, ST389). Notably, resistance determinants were especially common in high-risk clones, such as ST111, ST298, and ST235. These findings suggest that aminoglycoside resistance mutations occur spontaneously and independently, but that some resistance determinants also spread by vertical or horizontal transmission.

### Genomic characterization of an isolate with multiple AMEs.

**FIG 3:**
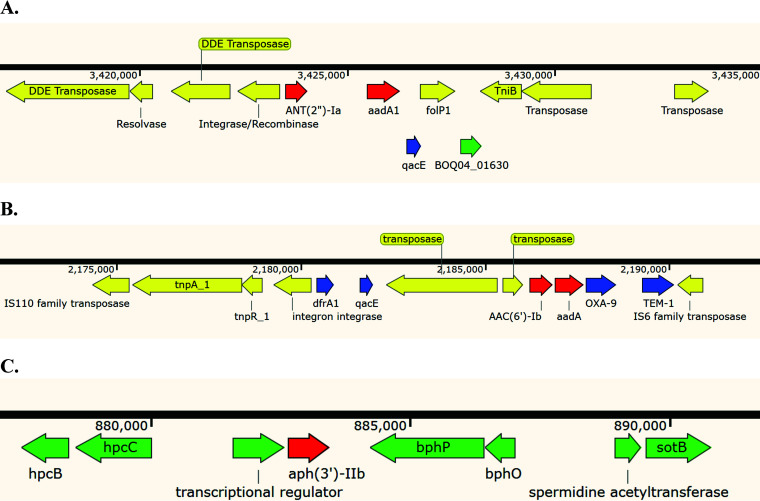
The context of AME genes present in PS1871. A. *ant(2″)-Ia* and *aadA1*. B. *aac(6’)-Ib* and *aadA1*. C. *aph(3′)-IIb*. Colors represent the following: red, AME genes; blue, genes conferring resistance to antiseptics and antibiotics other than aminoglycosides; yellow, transposition elements; green, other genes.

We next more closely examined the distribution of AME genes in our collection of isolates. As mentioned earlier, all isolates contained the *aph(3′)-IIb* gene, but most isolates did not contain additional AME genes. One exception was PS1871, an XDR isolate belonging to one of the more prevalent high-risk clones, ST235, and noted to have a total of five AME genes (*aph(3′)-IIb*, *aac(6’)-Ib*, *ant(2″)-Ia*, and two copies of *aadA1)*, as well as significant nonsynonymous mutations in *mexZ* (A155G, resulting in amino acid substitution of Y52C) and *armZ* (C610T resulting in amino acid substitution of R204C) (Table S1). To further characterize PS1871, we performed long-read sequencing and combined these results with the previously obtained short-read sequences to obtain its complete genome. This genome was used to identify the locations of the AME genes (Fig. 3). *ant(2″)-Ia* and *aadA1* are located among a cluster of transposon/insertion sequences, alongside *qacE* (encoding an efflux pump associated with antiseptic resistance). *aac(6’)-Ib* and a second copy of *aadA1* are located adjacent to two β-lactamase genes, *oxa-9* and *tem-1*, and near a different copy of *qacE* as well as *dfrA1*, a gene encoding a trimethoprim-resistance-conferring dihydrofolate reductase. These genes are also associated with a large transposon/insertion sequence complex. Finally, *aph(3′)-IIb* was located in a distinct region of the chromosome and was not adjacent to known AMR or transposase genes, consistent with it being part of the core genome of P. aeruginosa.

## DISCUSSION

We examined aminoglycoside resistance in P. aeruginosa bloodstream isolates over time at our institution. The susceptibility rates to amikacin and tobramycin were relatively high (94-95% and 86–88%, respectively) and comparable to those observed in the global SENTRY surveillance study (96% and 92%, respectively) (44). Susceptibility to gentamicin was lower and more variable, ranging between 73% and 87%. Susceptibility rates to aminoglycosides were relatively stable from 1999 to 2019, as was also observed in the SENTRY study. Similar trends were observed with several other commonly used anti-pseudomonal antibiotics. Of note, P. aeruginosa resistance rates and trends differ from study to study, suggesting institutional and regional variability perhaps influenced by local stewardship efforts. For example, Yayan and colleagues noted a significant increase in the prevalence of MDR isolates in a sample of 168 P. aeruginosa pneumonia isolates from 2004–2014 (45). On the other hand, the CDC estimated that antimicrobial stewardship efforts led to a 29% drop in MDR P. aeruginosa infections in the United States between 2012 and 2017 (46). Consistent with this, Abbara and colleagues studied time periods before (2007-2010) and after (2011-2014) an antimicrobial stewardship program intervention and found significant improvements in antibiotic usage and dramatic decreases in P. aeruginosa resistance rates, including a drop in resistance to amikacin from 30.9% to 0.8% (47). The antimicrobial stewardship program at NMH began in the 1990s but was substantially enhanced in 2006 with formal funding for both physician and pharmacist efforts.

Our genomic analyses indicated AME genes are relatively rare at NMH. The only exception was *aph(3′)-IIb*, although it does not provide resistance to the clinically relevant aminoglycosides gentamicin, tobramycin, or amikacin. In agreement with prior reports showing that *aph(3′)-IIb* is ubiquitous in P. aeruginosa (49), we found this chromosomal aminoglycoside phosphotransferase in every isolate in our collection. Other relatively common AME genes were *ant(2″)-Ia*, *aadA6*, and *aadA10* (6%, 4%, and 4% of isolates, respectively). Notably, *aadA* genes encode members of the ANT(3′)-Ia family of adenylating AMEs, which are the most common ANT AME genes and are typically found in integrons, plasmids and transposons. These AMEs primarily confer resistance to spectinomycin and streptomycin, but not gentamicin, tobramycin, or amikacin (9). Excluding *aph(3′)-IIb*, the overall prevalence of isolates containing AME genes was 14%, less than that reported in several other surveys. A French study of 120 P. aeruginosa bacteremia isolates collected between 1999 and 2004 showed that 25 (21%) samples contained one or more of the AME genes *ant(2″)-I*, *aac(6’)-I*, and *aac*(3)-*I* (50). In northeastern Poland, a study of 25 P. aeruginosa samples collected from two intensive care units between 2002 and 2009 detected *ant(2″)-Ia* in 36%, *aac(6’)-Ib* in 28%, and *aph(3′)-Ib* in 8% of the isolates (51). Our results may differ from these other studies because AME gene prevalence may vary from region to region and because the cited reports examined different types of isolate collections, including ICU isolates and non-bloodstream isolates.

In addition to the horizontal transfer of AME genes, P. aeruginosa strains may be resistant to aminoglycosides by acquiring mutations. The best studied of these types of mutations are those in the *mexZ* gene, which encodes the repressor of the MexXY-OprM efflux pump (11). Although these pumps are made by most P. aeruginosa isolates, resistance becomes pronounced when MexZ is inactivated (43, 52). *mexZ* mutations are relatively common in P. aeruginosa isolates cultured from the airways of patients with CF, perhaps because of frequent administration of inhaled tobramycin (53–55). However, *mexZ* mutations have also been reported in P. aeruginosa isolates cultured from non-CF patients. For example, Guenard and colleagues examined 57 non-CF isolates selected for their resistance to aminoglycosides and found that 77% contained mutations in *mexZ* [47]. In the current study, 21% of the isolates contained *mexZ* mutations predicted to disrupt the function of MexZ. Additional experiments are needed to definitively determine which of these *mexZ* mutations affect expression of *mexXY-oprM* and resistance to aminoglycosides.

Mutations in several other genes and loci have been implicated in aminoglycoside resistance, including *fusA1*, *parR*, *parS*, and *armZ* (12, 13, 15, 41, 42). In our collection of isolates, mutations in *parR*, *parS*, and *armZ* were individually associated with gentamicin resistance, and mutations in *fusA1* showed a trend toward an association. In contrast, only *armZ* mutations achieved a statistically significant association with tobramycin resistance. These findings confirm previous reports implicating *parR*, *parS*, and *armZ* in aminoglycoside resistance.

Prediction of aminoglycoside phenotypes from genotypes in P. aeruginosa has been challenging. Freschi and colleagues examined 33 P. aeruginosa isolates acquired from cystic fibrosis (CF) patients and seven reference genomes (48). They found poor correlations between genetic resistomes and phenotypic susceptibilities with the exception of fluoroquinolones. Nonetheless, our comprehensive regression analysis indicated associations between phenotypic gentamicin resistance and the presence of gentamicin-active AME genes as well as significant mutations in *mexZ*, *parS*, and *fusA1.* Associations with phenotypic resistance to tobramycin were limited to tobramycin-active AME genes, perhaps reflecting the smaller number of tobramycin nonsusceptible isolates versus gentamicin-nonsusceptible isolates in our collection (31 versus 45).

We examined in detail the genome of one XDR isolate, PS1871. Of the five AME genes present in this isolate, four occurred in pairs, and each pair was found in the context of transposon/insertion sequence genes. Additionally, the pairs were near *qacE* efflux pump genes, and one pair was located next to β-lactamase genes *OXA-9* and *TEM-1*. These findings suggest that these four AME genes are capable of being disseminated by horizontal transfer and that this process may be driven by exposure to antibiotics and disinfectants.

A limitation of our study is that we did not measure expression of AME genes, and it is possible that some of these genes were “silent.” Still, the presence of these genes was highly associated with aminoglycoside resistance, suggesting that they are being expressed. Likewise, we did not perform transcriptional or functional studies to determine which mutations actually disrupted protein function and affected gene expression. We did, however, use the PROVEAN program to estimate the likelihood of this occurring. In future studies, consideration of these additional factors may allow more accurate prediction of aminoglycoside resistance phenotypes.

## MATERIALS AND METHODS

### Antimicrobial susceptibility testing.

We obtained P. aeruginosa bloodstream isolates from three archived NMH collections cultured from 1999–2002, 2003-2009, and 2017–2019. All isolates were collected in accordance with and approval by the Northwestern University Institutional Review Board, which deemed that consent was not necessary. Susceptibility testing was performed by either the Vitek 2 platform (bioMérieux, Marcy l'Etoile, France) or broth microdilution, as previously described (19). Susceptibility versus nonsusceptibility (the latter defined as either resistant and intermediate classification) was determined using Clinical and Laboratory Standards Institute (CLSI) breakpoints (20).

### Whole genome sequencing.

The genome sequences of the 1999–2002 isolates were previously published (21–23). For the remaining isolates, bacteria were cultured and underwent DNA extraction as previously described (21). Library preparations were performed using the Nextera XT DNA Preparation Kit (Illumina, Inc., San Diego, CA, USA) following the manufacturer’s protocol. Short-read whole-genome sequencing was performed for all isolates using either the Illumina HiSeq or MiSeq platforms to generate paired-end reads. Reads were trimmed using Trimmomatic (v0.36). Draft genomes were assembled from trimmed paired-end reads using SPAdes (v3.9.1) and were filtered to remove contigs shorter than 200 bp, with less than 5-fold mean read coverage, or with alignment to phiX. For strain PS1871, additional long-read sequencing was performed using the Nanopore MinION platform, and hybrid assembly with short-read Illumina sequences was performed as previously described (24). Briefly, long-read sequencing libraries were prepared from unsheared genomic DNA using ligation sequencing kit SQK-LSK109 (Oxford Nanopore, UK) and sequenced on the MinION platform using a FLO-MIN106 flow cell. Guppy v3.4.5 was used to base call reads with the R9.4.1 high-accuracy model and to perform read quality filtering based on Q scores, demultiplexing, and barcode trimming. Assembly of Nanopore reads was performed using Flye v2.8.1 to generate a single circularized contig (25). Illumina reads were aligned to the assembly using BWA v0.7.17 (26), and assembly errors were corrected using Pilon v1.23 (27) with a minimum depth setting of 0.1. Serial read alignment and Pilon correction were performed sequentially until no further assembly corrections were generated. Custom software (Pilon Tools v0.1; https://github.com/egonozer/pilon_tools) was used to identify, manually assess, and correct any residual homopolymer assembly errors.

### Phylogenetic analysis.

Illumina read sequences were aligned to the reference genome sequence of PAO1 (AE004091.2) using bwa v0.7.15. Single nucleotide variants relative to the reference were identified using bcftools v1.9 using a haploid model and filtering read alignments with base quality lower than 25 or alignment quality less than 30. Variants were further filtered using the bcftools filter software (https://github.com/egonozer/bcftools_filter) to remove variants with single nucleotide variant (SNV) quality scores less than 200, read consensus less than 75%, read depths less than five, less than one read in both directions, or located within repetitive regions (as defined by blast alignment of the reference genome sequence against itself). For the total set of all consensus sequences, variant positions with base calls in less than 100% of isolate sequences (i.e., non-core positions) were masked with N’s using ksnp_matrix_filter.pl. Maximum likelihood phylogenetic trees were generated from the core genome alignments with FastTree v2.1.11. Phylogenetic trees were visualized using R v4.2.2 with ggtree package v3.7.1.

### Detection and contextualization of antibiotic resistance genes.

Assembled genome sequences were analyzed using the AMRFinderPlus tool (version 3.9.3, last accessed November 23, 2020; https://www.ncbi.nlm.nih.gov/pathogens/antimicrobial-resistance/AMRFinder/) for detection of AMR genes and resistance-associated alleles (28). The DDBJ Fast Annotation & Submission Tool (DFAST; v.1.2.4, last accessed January 3rd, 2021) was used to annotate genomic features of the PS1871 chromosome (29). SnapGene Viewer (from Insightful Science; available at snapgene.com) was used to generate open-reading-frame maps of the regions of PS1871 surrounding AME genes, and the results from DFAST were used to annotate these open reading frames.

### Analysis of gene mutations.

The sequences of the *mexZ*, *fusA1*, *parR*, *parS*, and *armZ* genes from P. aeruginosa strain PAO1 were obtained from https://pseudomonas.com (30) and used as references. Nucleotide BLAST (blastn) (31) was used to identify these genes in whole genome sequence assemblies of each isolate and extract the gene nucleotide sequences. The nucleotide sequences were aligned using MUSCLE v3.8.31. Identification of nonsynonymous variants, frameshift mutations, and premature stop codon mutations in each gene sequence relative to the PAO1 reference was performed using custom software (https://github.com/egonozer/fasta_alignment_gene_snps). Those nonsynonymous nucleotide variants with a high likelihood of disrupting protein function (scores < -2.5) were identified using the program PROVEAN (32, 33).

### Determination of Multilocus Sequence Type (MLST).

Each isolate’s multilocus sequence type (MLST) was determined using ResFinder (v4.1, last accessed November 23, 2020; Center for Genomic Epidemiology) (34, 35). MLSTs were assigned by ResFinder using PubMLST, an open-access curated database (36) that relies on a previously described MLST scheme for P. aeruginosa utilizing the seven housekeeping genes *acsA*, *aroE*, *guaA*, *mutL*, *mutD*, *ppsA*, and *trpE* (37). For the purposes of this study, those isolates having the same MLST as one of the 10 most globally prevalent high-risk clones listed by Del Barrio-Tofino and colleagues were designated as high-risk clones (4).

### Statistical analysis.

Statistical analyses were performed in Stata IC 17.0 (Statacorp LLC, College Station, TX). Changes in antibiotic susceptibility rates and changes in the number of isolates with at least one AME gene across each of the three time periods were analyzed using a test for trend (nptrend) and as a logistic regression comparing differences in the three time periods (i.e., 1999-2002; 2003-2009; and 2017–2019), where the referent time period was 2003–2009. For phenotypic aminoglycoside resistance predictions based on the presence of single AME genes, logistic regressions were performed using the presence of each individual AME gene. The presence of at least one AME gene predicted to have activity against the aminoglycoside of interest was also tested as a composite variable. AME genes predicted to have activity against gentamicin were *aac*(3)-*Id*, *aac*(3)-*IIIb*, *aac(6’)-Ib’*, *aac(6’)-II*, *aac(6’)-IIc*, and *ant(2″)-Ia.* AME genes predicted to have activity against tobramycin were *aac*(3)-*IIIb*, *aac(6’)-Ib*, *aac(6’)-Ib’*, *aac(6’)-II*, *aac(6’)-IIc*, and *ant(2″)-Ia*. AME genes conferring resistance to amikacin were not detected in our collection of isolates and were not considered further. For predicting categorical resistance, logistic regression was performed in a stepwise fashion with *P* < 0.2 used for removal from the model. Additionally, we completed separate logistic regression analyses to predict categorical resistance using the gentamicin-active and the tobramycin-active composite gene groups and the presence of *mexZ*, *fusA1*, *parR*, *parS*, or *armZ* premature stop codon mutations, frameshift mutations, and non-synonymous nucleotide substitutions predicted to impact protein function. *P*-values less than 0.05 were considered statistically significant.

### Data availability.

Whole genome sequences were deposited in the National Center for Biotechnology Information (NCBI) database. Accession numbers are listed in Table S1.
